# A retrospective study of clinical features of cough variant asthma in Chinese adults

**DOI:** 10.1186/s13223-019-0318-5

**Published:** 2019-01-17

**Authors:** Weiping Liu, Huaping Chen, Dehua Zhang, Feng Wu, Liqin Zhou

**Affiliations:** 1 Department of Respiratory Medicine, Huizhou Third People’s Hospital, Guangzhou Medical University, 1# Xuebei Avenue, Huizhou, 516002 Guangdong China; 2 Department of Reproductive Medicine, Huizhou Central People’s Hospital, National Sun Yat-sen University, Huizhou, Guangdong China

**Keywords:** Cough variant asthma (CVA), Classic asthma (CA), Induced sputum eosinophils, Pulmonary function, Airway hyperresponsiveness (AHR)

## Abstract

**Background:**

Clinical features of cough variant asthma (CVA) in Chinese adults are largely uncertain.

**Methods:**

A total of 303 patients newly diagnosed as uncontrolled asthma (symptom control and future risk of adverse outcomes), including 175 CVA and 128 classic asthma (CA), were enrolled in this retrospective survey. Clinical features including basic characteristics, pulmonary function, airway hyperresponsiveness (AHR) and cell counts of induced sputum, were compared retrospectively. All patients were classified into four inflammatory subtypes based on the counts of induced sputum eosinophils and neutrophils as eosinophilic (E), neutrophilic (N), mixed granulocytic (M), and paucigranulocytic (P) subtypes. Inflammatory subtype distribution was also compared.

**Results:**

Compared with CA patients, CVA patients were younger (P = 0.009), had a higher prevalence of female patients (P = 0.001), higher parameter values of baseline pulmonary function (P ≤ 0.01 for all), shorter duration of disease (P = 0.002), lower AHR (P = 0.001) and lower sputum eosinophil% (P = 0.009). There was a difference in the AHR distribution as the percentage of moderate and severe AHR in CVA was significantly lower than in CA (41.72% VS 64.70%, P = 0.001). The inflammatory subtype distribution was different as the proportion of E and M subtypes in CVA was lower than in CA (56.0% vs 67.19%, P = 0.049). The proportion of subtype P was the lowest and subtype M was the highest in both CVA and CA patients. There was a similar negative correlation of sputum eosinophil% with AHR in CVA and CA (r = − 0.337, P < 0.0001 and r = − 0.27, P = 0.026, respectively), and a positive correlation between sputum eosinophil% and improvement rate of FEV_1_ after inhalation of bronchodilator (ΔFEV_1_%) (r = 0.33, P = 0.01).

**Conclusions:**

CVA patients showed a better pulmonary function and lower airway inflammation in contrast to CA patients, which may participate in the pathogenesis of chronic cough in CVA.

## Background

Many patients in clinics are suffering from a chronic cough, which is defined as cough being the sole or predominant symptom lasting for at least 8 weeks, with absence of pulmonary disease [1]. Chronic cough is one of the commonest symptoms for which adults seek medical care worldwide. Studies have shown that CVA is one of the most common causes of chronic non-productive cough, as well as gastroesophageal reflux-associated cough, upper airway cough syndrome (UACS) and eosinophilic bronchitis (EB)-induced cough [2–4]. A prospective and multicenter survey in 2015 demonstrated that nearly one-third of chronic cough was associated with CVA in China [5], which was higher than that reported in western countries. However, CVA is easily overlooked and misdiagnosed clinically, since it presents with only cough but no wheezing as in CA.

Airway inflammation in asthma is heterogeneous. It is important to fully understand its pathophysiology and clinical phenotypic characteristics for the investigation of pathogenesis and development of treatment strategies. Nevertheless, as we know, the majority of current researches are relevant to classic asthma, and non-typical asthma patients are often excluded. Studies of CVA are few and the sample size is small [6–9]. Therefore, we conducted a retrospective study with a larger sample size to investigate: (1) the differences in clinical features between CVA and CA patients, by analyzing basic characteristics, pulmonary function, AHR, and cell counts of induced sputum; (2) the correlation between sputum eosinophil% and AHR or airway reversibility.

## Methods

### Subjects

Newly diagnosed asthma patients who visited respiratory clinics of Affiliated Third People’s Hospital of Guangzhou Medical University in Huizhou, were retrospectively included in this study from June 2016 to December 2017. All subjects were at uncontrolled stage. The level of asthma control was defined by asthma symptoms control (in the past 4 weeks, has the patient had daytime asthma symptoms more than twice/week and/or any night waking due to asthma and/or any activity limitation due to asthma and/or reliever needed for symptoms’ more than twice/week) and future risk of adverse outcomes (in the past 4 weeks, has the patient had FEV_1_% less than normal predicted value and/or any severe exacerbation due to asthma). Uncontrolled asthmatic patient had equal to or greater than 3 features as the above.

According to the 2018 GINA Guidelines of Asthma Management and Prevention [10], we divided all subjects into CVA group and CA group based on clinical symptoms and AHR test or bronchodilator reversibility test. CVA was diagnosed according to the following criteria: patients had a clinical history of a persistent cough (longer than 8 weeks) and AHR, but had no wheezing or dyspnea in contrast to CA, bronchodilators were effective against their coughs, excluding other diseases that caused chronic cough, such as gastroesophageal reflux, UACS, EB, taking angiotensin-converting enzyme inhibitors, etc. Patients with chronic cough and sputum productive of eosinophils who improve with inhaled corticosteroids (ICS) are often labelled as having EB. However, EB has no airway hyperresponsiveness as in CVA. All CVA subjects collected were tested with positive airway responsiveness, therefore, EB patients could be excluded from this study. CA was diagnosed based on the evidence of recurrent wheezing, shortness of breath, with or without chest tightness or cough, and reversible airway obstruction or AHR.

All subjects included had not received anti-asthma therapy before this study (including oral or inhaled corticosteroid, leukotriene receptor antagonists, antihistamines, etc.), and had no past history of upper respiratory tract infection in the last month. Result of chest radiograph or chest computed tomography was normal, excluding lung diseases (pneumonia, lung cancer, pulmonary tuberculosis, etc.). All subjects were over 17 years old, and had the cognitive competence to pass all the medical examinations under the guidance of doctors.

### Study design

Each subject underwent a clinical history inquiry, physical examination and imaging examination (chest radiography or computed tomography), pulmonary function test (PFT), AHR test or bronchodilator reversibility test, and finally sputum induction on the same day.

### PFT and AHR test

PFT, methacholine challenge test (AHR test), and bronchodilator reversibility test were performed with a computerized spirometer (MS-pneumo + aps, Jaeger, German) by professional technicians according to the 2014 Guideline for Pulmonary Function of Chinese Respiratory Society [11]. The forced expiratory volume in 1 s (FEV_1_), forced vital capacity (FVC), FEV_1_/FVC ratio, maximal mid-expiratory flow (MMEF) and maximum forced expiratory flow (MEF) were recorded in PFT. The degree of central airways obstruction was divided into four levels based on FEV_1_% predicted value: mild (≥ 70%), moderate (60–69%), between moderate and severe (50–59%), severe (35–49%), extremely severe (< 35%) [11].

AHR test was performed after PFT. Patients were asked to inhale gradually increasing doses of methacholine (0.9% saline, 0.078, 0.312, 1.125 and 2.504 mg), and FEV_1_ was measured after each inhalation. This test was stopped until a reducing in baseline FEV_1_ of 20%. AHR was calculated by PD_20_-FEV_1_ (cumulative dose of methacholine required to achieve a 20% decrease in FEV_1_) and defined by PD_20_-FEV_1_ ≤ 2.504 mg. AHR was divided into four levels based on PD_20_-FEV_1_ value: very mild (1.076–2.504 mg), mild (0.294–1.075 mg), moderate (0.035–0.293 mg), severe (< 0.035 mg) [11].

### Bronchodilator reversibility test

This test was performed on patients whose FEV_1_% predicted value < 70% in PFT. Patients were required to inhale 400 μg salbutamol through an inhalation device, and repeated the aforementioned PFT after 20 min. The result was defined as positive (reversible airflow obstruction) if postbronchodilator FEV_1_ increased ≥ 12% and absolute value increased ≥ 200 ml of prebronchodilator, moreover, postbronchodilator FEV_1_/FVC < 70% [11].

### Sputum induction and sputum cell counts

Before inducing sputum by the ultrasonic atomizer, patients were required to inhale 400 μg salbutamol. According to an established method [12], patients were asked to slowly inhale a single concentration of 3% hypertonic saline for 30 min, then rinsed the mouth with water, coughed deeply, and expectorated sputum into a sterile container. The opaque and high-density part of sputum sample was collected and diluted with 0.1% dithiothreitol in a water bath (37 °C) for 15 min, vortexed 1 min, and filtered through a 300 mesh fiber. After centrifuged at 3000 r/10 min, sputum cells were collected to prepare a Hematoxylin–Eosin-stained smear for the cells classification. Cells were counted with the percentage of 400 sputum cells under the microscope. A sputum sample was regarded as qualified for analysis when squamous epithelial cell contamination was < 10% and viable cell was > 80%.

### Statistical analysis

All data were analyzed using SPSS version 19.0 (USA) and P < 0.05 was considered of statistical significance. Continuous variables were expressed as mean (SD) depending on normal distribution or median (interquartile range) for non-normal distribution, while classification variables analyzed with Chi square test were expressed as number (percentage). Two groups of continuous variables were compared with T-test (normal distribution) or Mann–Whitney U test (non-normal distribution). Correlation between sputum eosinophils% and AHR or bronchodilator reversibility was analyzed by Spearman rank correlation.

## Results

### Differences in basic features and pulmonary function between CA and CVA patients

Of the 200 subjects in CVA and 140 subjects in CA enrolled, twenty-five CVA and twelve CA patients were excluded due to failed sputum induction. 175 CVA subjects (63 males and 112 females, all received AHR test) and 128 CA subjects (71 males and 57 females, 68 subjects received AHR test and 60 subjects received bronchodilator reversibility test) were included in this analysis, both with the ages ranged from 17 to 84 years old. CVA subjects consisted of young adults (17–39 years, n = 79), middle-age adults (40–59 years, n = 54) and elderly adults (60–79 years, n = 42). CA subjects consisted of young adults (17–39 years, n = 29), middle-age adults (40–59 years, n = 60) and elderly adults (60–79 years, n = 39).

As shown in Tables 1 and 2, CVA patients were significantly younger (P = 0.009) and presented a significantly higher prevalence of female (64% vs 44.5%, P = 0.001), as well as a shorter duration of disease (P = 0.002) than CA patients. In addition to wheezing, nearly 80% of CA patients had a history of chronic cough. All indices of pulmonary function in CVA were significantly higher than in CA (P < 0.01 for all).

**Table 1:** Basic features of asthma patients

Data	CVA (n = 175)	CA (n = 128)	P
Age (year)	46.25 (17.30)	51.34 (14.81)	0.009
Female, n (%)	112 (64)	57 (44.5)	0.001
Smoker, n (%)	30 (17.14)	25 (19.53)	0.594
Chronic cough, n (%)	175 (100)	101 (78.91)	0.0002
Duration (month)	26.21 (51.06)	61.64 (97.18)	0.002
Height (cm)	157.22 (12.82)	159.56 (8.44)	0.057
Weight (kg)	59.68 (11.57)	61.12 (9.55)	0.248
Body mass index (BMI, kg/m^2^)	23.33 (3.78)	23.86 (3.52)	0.23

Data are expressed as mean (SD) or number (percentage)

*CVA* cough variant asthma, *CA* classic asthma

**Table 2:** Pulmonary function of asthma patients

Data	CVA (n = 175)	CA (n = 128)	P
FEV_1_ (L)	2.28 (0.72)	1.72 (0.62)	< 0.0001
FVC (L)	3.01 (0.92)	2.73 (0.79)	0.006
FEV_1_% predicted value	87.75 (11.49)	65.69 (20.97)	< 0.0001
FVC % predicted value	97.36 (13.39)	85.25 (18.32)	< 0.0001
FEV_1_/FVC (%)	74.77 (9.65)	61.25 (12.95)	< 0.0001
PEF % predicted value	88.89 (15.54)	66.76 (21.70)	< 0.0001
MMEF (L/sec)	1.83 (0.92)	1.03 (0.63)	< 0.0001
MEF_50%_ % predicted value	56.53 (20.79)	32.36 (19.00)	< 0.0001
MEF_25%_ % predicted value	48.50 (23.78)	28.95 (18.12)	< 0.0001

Data are expressed as mean (SD)

*CVA* cough variant asthma, *CA* classic asthma, *FEV*_*1*_ forced expiratory volume in one second, *FVC* forced vital capacity, *PEF* peak expiratory flow, *MMEF* maximal mid-expiratory flow, *MEF25%* maximum forced expiratory flow at 25% FVC, *MEF50%* maximum forced expiratory flow at 50% FVC

### Differences in distribution of AHR between CA and CVA patients

The AHR of CVA was significantly lower than CA (P = 0.001). There was a difference in the AHR distribution as the percentage of moderate and severe AHR in CVA was significantly lower than in CA (41.72% VS 64.70%, P = 0.001) (Table 3).

**Table 3:** Distribution of AHR in asthma patients

Data	CVA (n = 175)	CA (n = 68)	P
PD_20_-FEV_1_ (mg)	0.73 (0.76)	0.42 (0.61)	0.001
Level of AHR			
Very mild, n (%)	37 (21.14)	8 (11.76)	0.001
Mild, n (%)	65 (37.14)	16 (23.54)	
Moderate, n (%)	53 (30.29)	25 (36.76)	
Severe, n (%)	20 (11.43)	19 (27.94)	

Data are expressed as mean (SD) or number (percentage); the proportion of moderate and severe AHR was compared with Chi square test

*CVA* cough variant asthma, *CA* classic asthma, *AHR* airway hyperresponsiveness

### Differences in induced sputum cell counts and inflammatory subtype distribution between CA and CVA patients

Sputum eosinophil% in CVA was significantly lower than in CA (P = 0.009), while an opposite result was showed in the macrophage% (P = 0.0004). No significant difference in sputum neutrophil% (P = 0.128) or lymphocyte% (P = 0.058) was found.

According to induced sputum eosinophil% (eos) and neutrophil% (neu), all patients were classified into four subtypes: eosinophilic (E) (eos% ≥ 2.5, neu% < 65), neutrophilic (N) (eos% < 2.5, neu% ≥ 65), mixed granulocytic (M) (eos% ≥ 2.5, neu% ≥ 65) and paucigranulocytic (P) (eos% < 2.5, neu% < 65) subtypes. There was a difference in the inflammatory subtype distribution as the proportion of E and M subtypes in CVA was lower than in CA (56.0% vs 67.19%, P = 0.049). The proportion of subtype P was the lowest and subtype M was the highest in both CVA and CA (Table 4).

**Table 4:** Results of induced sputum cell counts and inflammatory subtypes

Data	CVA (n = 175)	CA (n = 128)	P
Cell counts			
Eosinophil (%)	3.26 (12.78)	5.24 (19.08)	0.009
Neutrophil (%)	77.14 (34.57)	80.8 (31.55)	0.128
Macrophage (%)	10.79 (22.87)	6.35 (13.89)	0.0004
Lymphocyte (%)	0.64 (1.48)	0.56 (1.14)	0.058
Inflammatory subtypes			
Eosinophilic, n (%)	42 (24.0)	32 (25.0)	
Mixed granulocytic, n (%)	56 (32.0)	54 (42.19)	0.049
Neutrophilic, n (%)	54 (30.86)	41 (32.03)	
Paucigranulocytic, n (%)	23 (13.14)	1 (0.78)	

The results of cell counts are expressed as median (interquartile range) and compared with Mann–Whitney U test

Inflammatory subtypes are expressed as number (percentage) and compared with Chi square test

*CVA* cough variant asthma, *CA* classic asthma

### Correlation between sputum eosinophil% and AHR or airway reversibility

**Fig. 1:**
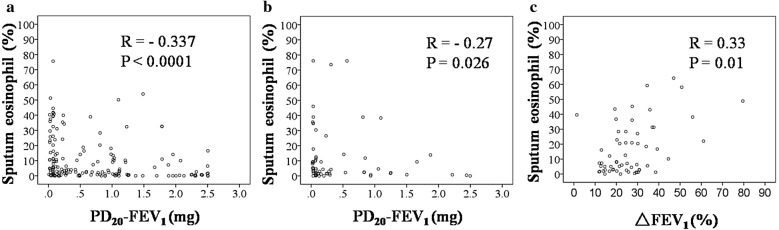
Correlation between sputum eosinophil% and PD_20_-FEV_1_ in CVA patients (**a**) or in CA patients (**b**). Correlation between sputum eosinophil% and the improvement rate in FEV_1_ after inhalation of bronchodilator in CA patients (**c**)

Significant and similar correlations were found between sputum eosinophil% and AHR in CVA and CA (r = − 0.337, P < 0.0001, and r = − 0.27, P = 0.026, respectively). There was also a significant correlation of sputum eosinophil% with the improvement rate in FEV_1_ after inhalation of bronchodilator (ΔFEV_1_%) in CA patients (r = 0.33, P = 0.01) (Fig. 1).

## Discussion

This is the first large-sample study on the clinical features of CVA patients in Chinese adults. With a broad range of all ages, a total of 175 CVA patients aged 17–84 years were evaluated in this study. Our study is superior to previous studies in such a broad range of all ages and a larger sample size. Therefore, the results of our study are reliable and may reflect the reality in practice.

This study retrospectively compared the baseline pulmonary function, AHR, and induced sputum cell counts in asthma patients. It showed that: (1) the parameter values of baseline pulmonary function in CVA, including indices of both central and peripheral airways obstruction, were significantly higher than in CA; (2) as with CA, eosinophilic airway inflammation (sputum eosinophil% ≥ 2.5) was also an important pathological feature of CVA; nevertheless, the AHR and eosinophilic airway inflammation of CA were significantly higher than those of CVA. These findings may be related to the mechanism of chronic cough without wheeze in CVA in contrast with CA. Some studies, with a small sample size, revealed that these factors, such as airflow obstruction at central airways, heightened cough sensitivity, higher wheezing threshold, lower AHR, prostaglandin-E_2_ and neuropeptide substance P secreted by eosinophils, mild airway remodelling, lower total IgE and smaller numbers of sensitized allergens [13–19] were considered to be associated with cough without wheeze in CVA. However, these possible mechanisms of chronic cough are controversial and the exact mechanism remains unclear. With a larger sample size and adults of all age levels involved, our study demonstrated that lower AHR, lower eosinophilic inflammation, and mild airflow obstruction were associated with presenting manifestations in CVA.

There is also a controversy on peripheral or central airways obstruction of CVA. We noticed that 51.43% of CVA subjects showed normal or almost normal pulmonary function (only with peripheral rather than central airways obstruction at baseline), and other subjects had a mild central airways obstruction (FEV_1_% predicted value ≥ 70%); while 87.5% of CA patients showed a central airways obstruction, and 56.2% of CA patients had moderate to extremely severe central airways obstruction (FEV_1_% predicted value ≤ 69%). These results indicate that there is no relationship between the site of airway obstruction (peripheral or central airways) and chronic cough mechanism of CVA.

Induced sputum cell counts, a convenient and non-invasive method, is considered more accurate in assessing airway eosinophilic inflammation when compared with fractional exhaled nitric oxide (FeNO) and peripheral blood eosinophil count. There is no unified classification standard for sputum cell counts in Chinese and Westerners. According to the definition of sputum eosinophil% ≥ 2.5 as eosinophilic inflammation and neutrophil% ≥ 65 as neutrophilic inflammation recommended in 2016 Chinese National Guidelines on Diagnosis and Management of Cough [20], asthma patients were classified into four cell subtypes, which provided more evidence for individualized treatment. ICS is effective for eosinophilic asthma, but poor for non-eosinophilic asthma. Our study found that 44% CVA and 32.81% CA patients were diagnosed as non-eosinophilic asthma; thus, these patients needed more treatment strategies to control airway inflammation, in addition to ICS.

Our study has some limitations. First, it was conducted in a retrospective fashion. Second, sputum neutrophil might increase by smoking cigarette, age and air pollution, while the sample size was significantly increased if smoking and non-smoking patients were included in this study.

## Conclusions

CVA patients showed higher parameter values of baseline pulmonary function, lower airway reactivity and airway eosinophilic inflammation in contrast to CA, which may correlate with the pathogenesis of chronic cough in CVA.

## Abbreviations

CVA: cough variant asthma
CA: classic asthma
FEV_1_: forced expiratory volume in one second
FVC: forced vital capacity
PEF: peak expiratory flow
MMEF: maximal mid-expiratory flow
PEF: peak expiratory flow
MEF25%: maximum forced expiratory flow at 25% FVC
MEF50%: maximum forced expiratory flow at 50% FVC
AHR: airway hyperresponsiveness
PFT: pulmonary function test
ΔFEV_1_%: the improvement rate in FEV_1_ after inhalation of bronchodilator
UACS: upper airway cough syndrome
EB: eosinophilic bronchitis
ICS: inhaled corticosteroids
